# The Medical and Social Aspects of Consumption

**Published:** 1911-07-29

**Authors:** 


### CONGRESSES OF THE WEEK.

### THE MEDICAL AND SOCIAL ASPECTS OF CONSUMPTION.

### A SUMMARY OF THE CONFERENCE RESULTS.

Although several of the papers read before the Conference held last week by the National Association for the Prevention of Tuberculosis covered old ground, there were many valuable criticisms presented for consideration, as well as useful suggestions. Dr. Ernest J. Schuster pointed out some of the weaknesses of the Insurance Bill in relation to the tuberculous. For example, that the Bill does not give a right to any particular class of persons, whether insured or not, to be received into any sanatorium, nor is it clear whether the County Council or other authority by whom a sanatorium is constructed will also be entrusted with the duty to manage and maintain it. Again, the provisions of the Bill do not enable any authority to compel the patients to submit to sanatorium treatment. Patients may decline to be admitted or may leave the institution after a very short stay. Dr. Schuster also considered that it is a pity that the Bill does not provide in a more definite manner for the support of hospital and district nurses. In Germany it is stated that the hospitals receive a great deal of support in consequence of the rules which enable a sick fund to compel a member in certain specified events to substitute treatment in a hospital for medical benefit and sick pay. The expenditure for this purpose in 1909 amounted to over two million pounds. Dr. Schuster was also rather severe upon the desire of the doctors that the patients should have free choice upon the question of their medical attendant. While he thinks that the inferior man is more often than not elected by a committee in a system of free choice of doctors, the man of deficient moral character would probably find the greatest favour. The man who knows how to curry favour with a committee also will know how to curry favour with the patients, especially when the receipts of insurance benefit depends upon a medical certificate. Again, it was pointed out that large classes of the population are left untouched by the Bill, more particularly all the children, all married women who are not in the regular service of an employer, all deposit contributors who have exhausted their deposit, all workers who work on their own account or for occasional employers. Some of these excluded classes live under the worst conditions and are of all the most in need of help. These and other criticisms are summarised in the strongly expressed opinion that there will be great need for voluntary effort in many directions, and without such voluntary aid the scheme will be "worse than useless." This applies not merely with regard to voluntary aid in the working of the scheme, but to financial aid also.

Mr. C. S. Loch read a paper on the "after-care" of cases which had been admitted into sanatoriums. Although the title of his paper was "After-care," the difficulty in many instances of getting patients into a sanatorium was referred to. It is apt to be forgotten that "a case of tuberculosis" is a "living" being, with all such a being's likes and dislikes and possibly difficulties of coming to a decision. He gave as an illustration that in one case thirty-seven letters were written, fourteen visits paid to the house, and twenty-seven calls made with regard to the matter by the patient's wife. Mr. Loch considers that in dealing with an illness like phthisis, work must be carried out through local agencies in comparatively small areas. The establishment in every district of a "dispensary for the prevention of consumption" he considers advisable. Associated with this dispensary it is necessary that there should be a group of co-operating and assisting friends, brought into association on charity organisation lines. In conclusion, Mr. Loch emphasised the point upon which Dr. Schuster laid stress, that both in the "before-care" and "after-care" of tuberculous cases, as the Bill stands, there is great need for charity.

Dr. H. W. McConnel also referred to the "after-care." The methods of work adopted for the patients in various sanatoriums was referred to. At some the patients are set to work at their own trades; at others endeavours are made to teach them some occupation which may be useful. At others, again, some work merely for the sake of obtaining graduated work is devised. After leaving the institutions Dr. McConnell mentioned that the Charity Organisation Society does much useful work in looking after the patients and in aiding them to obtain employment.

An interesting paper on the economic cost to the community of tuberculosis was read by Mr. Waldorf Astor. The German figures were quoted, which are stated to show that 46 per cent. of cases which have been in sanatoriums are still able to work five years after leaving the institution. Afterwards various figures to show the loss of wage-earning capacity yearly owing to deaths from tuberculosis were given.

The diagnosis of tuberculosis in persons under sixteen years of age at the hospitals and elsewhere was referred to by Dr. Sykes. He thinks that much might be done by philanthropic and municipal agencies by sending suspected cases to physicians at hospitals and asking them for a report.

Open-air recovery schools was the subject of a paper by Dr. Ralph Williams, and a list given of the nature of cases admitted to the school under his care. The diagnosis of nine cases of enlarged mediastinal glands, those accustomed to examine children would possibly consider to be a diagnosis open to question.

Miss McGaw also dealt with the need for tuberculosis schools. Schools of this nature no doubt have considerable value, but the percentages given by Miss McGaw of the frequency of tuberculosis in children, it is to be feared, cannot be taken as accurate.

The remainder of the papers dealt chiefly with various aspects of sanatorium treatment. The existing accommodation was dealt with by Dr. Nathan Raw. The value of sanatorium treatment by Dr. Arthur Latham and Dr. Jane Walker, and the educational value of the sanatorium by Dr. J. J. Perkins and Dr. Marcus Paterson. A paper upon the question of segregation of advanced cases of tuberculosis was read by Dr. Maxwell Williamson, and Dr. Squire dealt with the home treatment of consumptives under the National Insurance Bill.

The question of segregation is a difficult and important question. Dr. Williamson showed how Edinburgh is dealing with the matter. "A pavilion of the City's magnificent Infectious Hospital" is set apart for the exclusive use of patients suffering from the last stages of the disease. It was recognised that the success of any efforts of this kind must greatly depend upon the attractive nature of the hospital accommodation afforded. It was stated that sufferers and their friends have been quick to recognise the excellence of the accommodation provided. One rather pathetic recommendation is made. It is that a room should be set apart for patients during the last few days of life. This is no doubt necessary for the comfort and spirits of the living, yet to the patient removed what the removal indicates must in many instances be painful. Dr. Squire in his paper only referred very briefly to the bad cases which with advantage might be removed to an institution such as that described by Dr. Williamson. The cases in the mind of Dr. Squire appeared to be chiefly those cases not suitable for or unable to gain admittance to or be retained in a sanatorium, yet who are unable to work. For these general supervision of the home life is required. The Bill only provides them with medical attendance and 5s. a week, but efficient medical attendance and advice covers a wide field.

